# The effect of TCM triple rehabilitation and ear acupoint bean pressing on laparoscopic surgery for gastric cancer

**DOI:** 10.1097/MD.0000000000039423

**Published:** 2024-08-30

**Authors:** Guangzhuo Ren, Liping Yang

**Affiliations:** a Department of Nursing, Baoji Hospital of Traditional Chinese Medicine, Baoji, Shaanxi, China; b Department of Oncology, Baoji Hospital of Traditional Chinese Medicine, Baoji, Shaanxi, China.

**Keywords:** clinical effects, complications, gastric cancer, laparoscopic radical surgery, triple pre-rehabilitation nursing

## Abstract

To explore the clinical value of the triple pre-rehabilitation nursing model in laparoscopic radical surgery for gastric cancer. Eighty-two gastric cancer patients admitted to a certain hospital from May 2020 to May 2023 are included in this study. Patients were divided into control group (CG) and treatment group according to different nursing methods. Comparisons were made by comparing perioperative indicators, immune indicators, sleep quality, nutritional indicators, and anxiety before and after patient care, as well as whether or not to utilize the acupoint patch combined with ear acupoint bean pressing burial in Chinese medicine care. Under the conditions of the triple pre-rehabilitation nursing model, the observation group (OG) patients had their first exhaust time, first bed activity time, first bowel movement time, and hospitalization time of 62.15 ± 5.93, 18.67 ± 7.55, 2.05 ± 0.58, and 7.21 ± 1.05, respectively. The postoperative values of ALB in the CG and OG were 31.59 ± 7.65 and 36.08 ± 8.27, respectively, while the postoperative values of prealbumin were 0.19 ± 0.05 and 0.27 ± 0.09, respectively. The sleep quality of the CG before nursing was 22.57 ± 3.66, and after nursing was 14.36 ± 3.72. The satisfaction rate of the OG was 56.10%, while that of the CG was 46.34%. Patients can better adapt to the treatment process, reduce anxiety, and improve the treatment effect and quality of life after the triple pre-rehabilitation nursing care model and acupoint paste combined with ear acupoint bean pressing burrowing in traditional Chinese medicine nursing.

## 1. Introduction

Gastric cancer is a common malignant tumor, with high-incidence rate and mortality. According to statistics, the mortality rate of gastric cancer patients is the highest within 5 years, with a survival rate of <50%. The most commonly used treatment method for gastric cancer in clinical practice is radical gastrectomy, which has the characteristics of good recovery effect and high surgical success rate.^[1,2]^ The traditional treatment method for gastric cancer mainly involves surgical resection, but this method brings great physical and mental pressure to patients. In recent years, with the continuous development of laparoscopic technology, Laparoscopic Radical Gastrectomy for Gastric Cancer (LRG-GC) has gradually become an important means of treating gastric cancer. Compared with traditional open surgery, laparoscopic surgery has advantages such as less trauma and faster recovery.^[3,4]^ However, due to factors such as surgical trauma and postoperative complications, LRG-GC still faces some clinical difficulties. At the same time, gastric cancer can also have a significant impact on the physical and psychological well-being of patients, leading to feelings of fear and anxiety (F&A). The generation of these emotions can also affect the recovery effect of patients before, during, and after surgery, leading to physical disorders and even life-threatening situations.^[5,6]^ Therefore, in the treatment process of gastric cancer patients, effective nursing measures need to be taken to strengthen their dietary care, so as to provide the necessary nutrients for the body and ensure effective recovery. At present, many studies have focused on exploring the surgical techniques and efficacy of LRG-GC, but there is relatively little research on postoperative rehabilitation nursing models. Rehabilitation nursing is a comprehensive and systematic nursing model aimed at providing comprehensive support and assistance for patients to recover their health through multidisciplinary comprehensive interventions. The triple pre-rehabilitation nursing (TPRN) model is a new nursing model that has been fully developed and optimized by modern nursing. It provides personalized and comprehensive rehabilitation nursing services for patients by emphasizing comprehensive pre rehabilitation in 3 aspects: psychology, nutrition, and exercise. Through extensive application, it has achieved good therapeutic effects in the field of malignant tumors.^[7,8]^ Prior to LRG-GC, the TPRN model focused on guiding patients to engage in moderate to high-intensity aerobic and strength exercises, which can help improve their physical fitness and tolerance. In addition, this model also emphasizes nutritional support primarily based on protein supplementation, ensuring that patients receive sufficient nutrition before surgery to better cope with surgical expenses. In addition to physical preparation, psychological support is also an important part of preoperative pre rehabilitation. Anxiety and depression are common psychological issues among many surgical patients, which may affect their surgical outcomes and postoperative recovery. Through psychological support such as conversation and relaxation training, patients can eliminate anxiety and maintain a calm mindset to welcome surgery. Traditional rehabilitation measures usually start after surgery, but in reality, early preoperative rehabilitation can bring more benefits to patients with abdominal tumors.^[9,10]^ Research has shown that the TPRN strategy can significantly improve the perioperative functional ability of patients undergoing colorectal cancer surgery and promote postoperative functional recovery.^[11]^ Based on this, this study applies the TPRN model for training in perioperative management of LRG-GC, and explores its clinical effects on LRG-GC, providing reference and inspiration for clinical practice.

## 2. Materials and methods

### 2.1. General data

This retrospective study included gastric cancer cases enrolled in hospital from May 2020 to May 2023. Inclusion criteria: (1) meet the diagnostic criteria for gastric cancer; (2) it has indications of laparoscopic radical gastrectomy and can be tolerated; (3) the patient signs the informed consent. (4) The patient information is complete; exclusion criteria: (1) surgical contraindications; (2) distant metastasis and spread of tumor tissue; (3) combined with serious infection, anemia, etc; (4) there is coagulation dysfunction; (5) accompanied by serious heart, lung and other organ dysfunction; (6) use of drugs that affect the recovery of gastrointestinal function; (7) accompanied by epilepsy, schizophrenia and other psychiatric diseases; (8) communication difficulties, complete aphasia, hearing or intellectual impairment. (9) Loss of relevant information and loss of access. A total of 82 patients were included in this retrospective study. In this study, patients were divided into control group (CG) and observation group (OG) according to different nursing methods, with 41 patients in both groups. There are 23 males and 18 females in CG in the interval of 25 to 65 year-old with an mean age of 36.75 ± 5.8 years. There are 22 males and 19 females in the OG, with an average age of 37.02 ± 5.9 years, ranging from 25 to 65 years old. The difference between the above general information of the 2 groups of patients is not statistically significant (*P* > .05) and is comparable. In this clinical efficacy comparison trial of TPRN mode on LRG-GC, the inclusive and exclusive standard for cases are shown in Table 1. This project has been approved by the Medical Theory Committee of our hospital, and all guardians of patients have signed informed consent forms.

**Table 1:** Case inclusion and exclusion criteria.

Serial number	Standard	Standard content
1	Inclusion criteria	Confirmed diagnosis through gastroscopy before surgery
2		The surgical margin is negative
3		Not receiving neoadjuvant chemotherapy or radiation therapy
4		Normal heart, liver, and kidney functions
5		No preoperative movement disorders
6	Exclusion criteria	Patients who are unable to undergo laparoscopic surgery
7		Unable to cooperate in completing training
8		Malignant tumor distant metastasis
9		Individuals with consciousness disorders who are unable to express their condition
10		Individuals with abnormal coagulation function
11		Individuals with immune system disorders
12		Simultaneous resection of organs such as colon, liver, spleen, and pancreas is required

### 2.2. Methods

(1) CG: CG adopts conventional nursing methods, which are mainly divided into 7 parts, including: ① maintain the quietness and cleanliness of the ward, ensuring appropriate insulation and humidity. ② Introducing the layout, facilities, and hospital rules and regulations of the ward to patients can help them adapt to the hospital environment more quickly, improve medical service efficiency, ensure medical safety, and help establish a good doctor-patient relationship and hospital image. ③ Continuously observe and record the patient’s vital signs and changes in their condition. ④ Perform routine medication and infusion care procedures. ⑤ Postoperative education is necessary to monitor the patient’s exhaust, bowel movements, and adverse reactions Check if the indwelling catheter is securely fixed and record the amount, color, and nature of the drainage fluid. ⑦ Guide patients on lifestyle precautions and provide them with reasonable dietary and lifestyle recommendations.

(2) OG: In the experiment, a 7-day TPRN training was designed based on CG. Firstly, exercise care is used for gastric cancer patients, including aerobic and anaerobic exercise. Aerobic exercise includes jogging and brisk walking. Before each aerobic exercise, it is necessary to warm up for about 5 minutes, and maintain it 3 times within 7 days for 20 minutes each time. Anaerobic exercise for gastric cancer patients includes resistance exercise and respiratory training. Resistance exercise can be performed by climbing stairs (2–3 floors), which helps patients enhance their physical fitness. Respiratory exercise can be performed through deep breathing, holding breath, and other methods, which can help patients improve respiratory function and enhance lung muscle strength.^[12]^ These exercises can help patients enhance their cardiovascular function, improve their physical fitness, and prepare them for surgery. During exercise, it is important to pay attention to moderation and avoid excessive fatigue.

Next is psychological nursing training, and psychological intervention training plays an important role in LRG-GC. Nursing staff should provide proactive psychological care, strengthen communication and exchange with patients, identify their negative emotions, and take corresponding measures for psychological counseling. At the same time, it is necessary to assess the patients’ psychological condition and provide related counseling and treatment for those with anxiety and depression. Psychological counseling can establish good trust relationships, help patients understand themselves, and overcome psychological difficulties. Psychological therapy applies relevant theories and techniques to induce psychological, behavioral, and even physiological changes in patients. Specific measures include encouraging patients to express their inner feelings, reducing their fear of illness or surgery, providing psychological comfort, providing hospital routine and treatment information, answering patient questions, and guiding patients in self-regulation. In addition, to explain gastric cancer related knowledge and successful cases to patients and their families, seek family support, guide patients to participate in social activities, and use relaxation training, music therapy, etc to alleviate anxiety.^[13]^ Throughout the process, it is also very important to pay attention to rest and ensure sufficient sleep quality. These measures aim to help patients overcome psychological distress, enhance self-control and psychological resilience, and face illness and treatment with a positive attitude.

Finally, there is nutritional intervention, which requires a comprehensive nutritional assessment of the patient to understand their nutritional status, dietary habits, and intake. This study rated patients based on a nutritional risk screening questionnaire and developed personalized dietary standards for patients with scores <3. Increasing the intake of fruits, vegetables, and protein, and limit the intake of high-fat, high calorie foods. At the same time, it is necessary to avoid bad habits such as overeating and excessive drinking. For patients at risk of malnutrition, appropriate nutritional supplements such as protein powder, vitamin and mineral complexes can be considered. The selection and use of supplements should follow the advice of a doctor and avoid blind self-use. After surgery, appropriate nutritional support can be provided to patients based on their recovery status. Adequate energy and nutrients can be provided through enteral or parenteral nutrition pathways to promote patient recovery. Regularly to monitor the nutritional and physical condition of patients, and promptly detect and manage malnutrition and related complications.^[14]^ Meanwhile, based on the monitoring results, it is necessary to adjust the nutritional intervention plan in a timely manner to ensure that the nutritional needs of patients are met.

In order to verify the effect of traditional Chinese medicine (TCM) care, the study added acupoint patch combined with ear acupoint bean pressing burrowing in the OG. Referring to “*New Hand Acupuncture Therapy*” written by Zhu Zhenhua, relevant acupoints such as spleen acupoint (located at the transverse line of the thumb interphalangeal joint on the palm side of the hand), large intestine acupoint (located at the transverse line of the 2nd knuckle joint of the index finger on the palm side of the hand), and gastrointestinal pain point (located at the line connecting the midpoint of the 3rd and 4th metacarpal bone gaps with the midpoint of the carpal transverse stripe) were selected. Nursing staff carried out acupressure combined with ear acupoint bean pressing burrowing on the patients after 5 hours postoperatively, and the whole acupressure combined with ear acupoint bean pressing burrowing started from the postoperative period to the cutoff 1 day before the patients were discharged from the hospital.^[15]^ In the process of specific Chinese medicine care to timely observe the patient’s condition, caregivers need to pay attention to hand hygiene disinfection work.

### 2.3. Observed indicator

(1) Perioperative observation indicators: Observe the wound anastomosis, infection, and complications of 2 groups of patients within 30 days after surgery and discharge.
(2) Gastrointestinal symptom score: This study used the Gastrointestinal Symptom Grading Scale as a scoring standard, scoring from 5 directions: bloating, abdominal pain, nausea, vomiting, and constipation. Each direction is scored using the Likert 4-level scoring method, with scores ranging from 0 to 20. Patients with higher gastrointestinal symptom scores indicate more pronounced gastrointestinal reactions. This scoring is evaluated and scored by nursing nurses at 6 hours and 72 hours after surgery.
(3) Immune indicators: Immunoglobulin is used as a specific indicator for the detection of immune indicators. Immunoturbidimetry is used to collect samples at the same time point, and then sample testing is carried out.
(4) Nutritional indicators: The study used serum albumin (ALB) and prealbumin (PAB) as indicators for nutritional testing. The bromocresol green method was used to detect serum ALB, and the rate scattering turbidity method was used to detect PAB.
(5) Gastrointestinal function recovery: Record the duration of postoperative abdominal pain and bloating, the time of first defecation and defecation, the time of bowel sounds recovery, and the time of discharge.
(6) Patient emotions: Use the Zong Self Rating Anxiety Scale and Zong Self Rating Depression Scale to evaluate the patient’s negative emotions within 30 days before and after nursing. The lower the score, the more severe their F&A is.
(7) Satisfaction: It is calculated through a self-made survey questionnaire conducted by our hospital, with a total score of 100 points. It has 3 levels: very satisfied (≥85 points), satisfied (70–84 points), and dissatisfied (<70 points). The total satisfaction rate = very satisfied + satisfied.

Perioperative observations is the primary outcome in the original article. The secondary outcome was gastrointestinal symptom score, nutritional indicators, immune indicators, gastrointestinal function recovery, patient emotions, satisfaction.

### 2.4. Statistical methods

This study uses SPSS 22.0 statistical software for all relevant statistical analyses. Measurement data are expressed as mean ± standard deviation, and count data are expressed as percentages (%). Baseline characteristics are compared using independent t-tests for continuous variables (e.g., age) and chi-square tests for categorical variables (e.g., gender), with normality and homogeneity of variances checked via the Shapiro–Wilk test and Levene test, respectively. The samples conform to a normal distribution. Pairwise comparisons of primary and secondary outcomes between the CG and OG use independent t-tests for continuous data and chi-square or Fisher exact tests for categorical data. Within-group comparisons (pre- and post-intervention) employ paired t-tests. For all statistical methods, a significance level of *P* < .05 is considered statistically significant.

## 3. Results

### 3.1. Basic information

By collecting and organizing clinical information of patients, a total of 5 indicators were collected, including height, weight, BIM, nutritional screening values, and age. All statistical information is shown in Table 2, and there is no significant difference (*P* > .05) between the 2 groups of patients in non-related disease data. The comparison showed strong comparability.

**Table 2:** Comparison of basic information.

Group	Number of patients/case	Age/year	Staging of disease course (I, II, III)	Nutrition screening value/score	BMI/kg/m^2^
Control group	Male 23, Female 18	36.75 ± 5.8	13, 16, 12	3.15 ± 0.91	22.67 ± 2.26
Observers	Male 22, Female 19	37.02 ± 5.9	15, 12, 14	3.16 ± 0.88	22.71 ± 2.34
*x*^2^/*t* value	/	0.26	0.965	0.89	0.591
*P*-value	/	.596	.511	.315	.602

### 3.2. Comparison of perioperative indicators

Table 3 shows the comparison results of the first exhaust time (FET), first time getting out of bed activity time (FGOBT), first bowel movement time (FBMT), and hospitalization time (HT) between 2 groups of patients during the perioperative period. Through comparison, it was found that the time of the 4 indicators in OG was significantly reduced compared to CG, and the differences were significant (*P* < .05).

**Table 3:** Comparison of perioperative indicators.

Group	Number of patients/case	FET/h	FGOBT/h	FBMT/d	HT/d
Control group	41	78.26 ± 9.12	29.83 ± 8.05	3.98 ± 0.93	9.57 ± 1.49
Observers	41	62.15 ± 5.93	18.67 ± 7.55	2.05 ± 0.58	7.21 ± 1.05
*x*^2^/*t* value	/	10.21	2.95	4.69	4.18
*P*-value	/	.0153	.0169	.0105	.0126

FBMT = first bowel movement time, HT = hospitalization time.

### 3.3. Gastrointestinal symptom score

**Figure 1.**
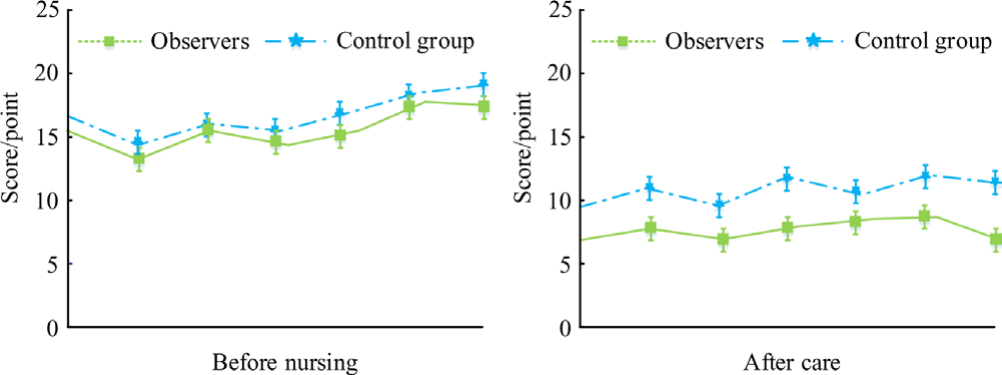
Comparison of gastrointestinal symptom scores.

Figure 1 shows the comparison of gastrointestinal symptom scores (GSS) between 2 groups of patients before and after TPRN. Before TPRN, there was *P* > .05 in GSS in both groups, and with *P* < .05 in GSS after TPRN.

### 3.4. Immune and exercise indicators

Table 4 shows the comparison results of immune and exercise indicators between 2 groups of patients before and after TPRN. Before TPRN, there was *P* > .05 in IgG and 6MWT between the 2 groups, while after TPRN, the IgG and 6MWT index values of OG were significantly higher than those of CG, with *P* < .05.

**Table 4:** Comparison of immune and exercise indicators.

Group	Number of patients/case	IgG (g/L)		6MWT (m)	
		Preoperative	Postoperative	Preoperative	Postoperative
Control group	41	13.05 ± 0.92	10.05 ± 0.88	575.27 ± 66.81	551.84 ± 59.87
Observers	41	12.93 ± 0.89	13.92 ± 0.93	576.11 ± 65.29	621.05 ± 61.69
*t*-value	/	0.51	2.81	0.43	2.27
*P*-value	/	.621	.0089	.701	.031

FET = first exhaust time, FGOBT = first time getting out of bed activity time.

### 3.5. Nutritional indicator

Table 5 shows the comparison results of nutritional indicators among patients before and after TPRN. Before TPRN, there was *P* > .05 in ALB and PAB. After TPRN, the ALB and PAB index values of OG were significantly higher than those of CG, and the ALB and PAB between the 2 groups were significant (*P* < .05).

**Table 5:** Comparison of application indicators.

Group	Number of patients/case	ALB (g/L)		PAB (g/L)	
		Preoperative	Postoperative	Preoperative	Postoperative
Control group	41	39.84 ± 10.92	31.59 ± 7.65	0.33 ± 0.12	0.19 ± 0.05
Observers	41	39.93 ± 11.21	36.08 ± 8.27	0.33 ± 0.11	0.27 ± 0.09
*t* value	/	0.026	1.963	0.486	5.946
*P*-value	/	.893	.0458	.619	.00595

### 3.6. Comparison of gastrointestinal function recovery results

Table 6 indicates the recovery time of gastrointestinal function before and after triple prehabilitation nursing care combined with acupoint plastering combined with ear acupoint bean pressing burying therapy in both groups. The duration of abdominal pain, bloating, and recovery time of bowel sounds in OG were significantly reduced compared to CG, with statistical significance (*P* < .05).

**Table 6:** Comparison of gastrointestinal function recovery.

Group	Number of patients/case	Duration of abdominal pain (h)	Duration of abdominal distension (h)	Recovery time of intestinal sounds (h)
Control group	41	12.59 ± 2.04	21.36 ± 3.27	36.41 ± 3.5
Observers	41	7.96 ± 1.58	9.21 ± 2.68	18.26 ± 2.79
*t* value	/	10.85	15.76	19.86
*P*-value	/	.00843	**.00458**	**.00249**

### 3.7. Comparison results of sleep quality

**Figure 2.**
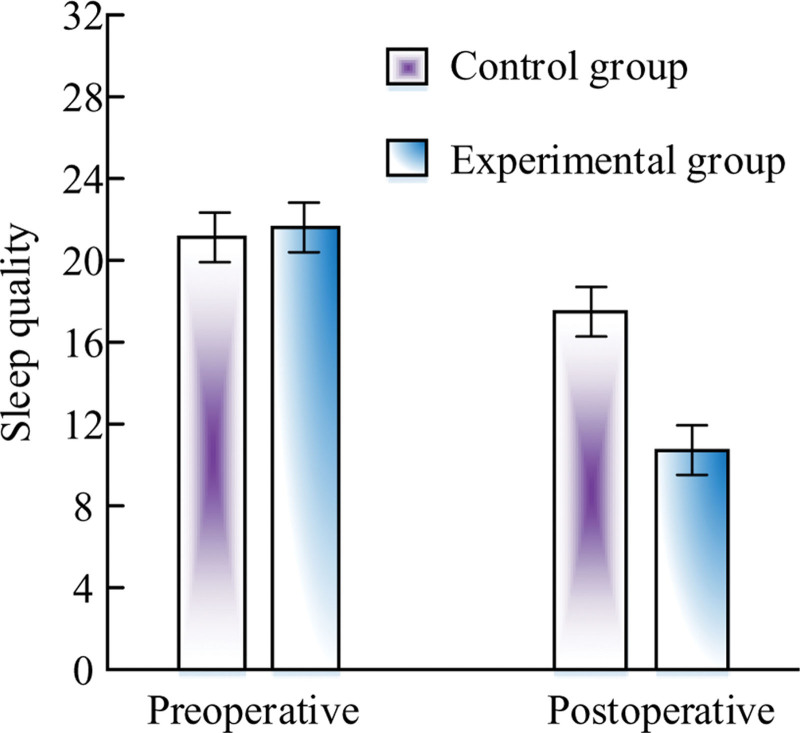
Comparison of preoperative and postoperative sleep quality results.

Figure 2 shows the comparison of sleep quality between preoperative and postoperative patients. Before TPRN, there was no statistically significant difference in sleep quality between the 2 groups (*P* > .05). After TPRN, the sleep quality of OG was significantly higher than that of CG, and the sleep quality between the 2 groups was statistically significant (*P* < .05).

### 3.8. Patient’s emotional comparison results

Table 7 shows the comparison results of sleep quality between preoperative and postoperative patients, and there is no obviously significant change in patient emotions before surgery (*P* > .05). After surgery, OG utilized TPRN, and the improvement in patient emotions was significantly higher than CG. The emotional changes between the 2 groups were statistically obvious (*P* < .05).

**Table 7:** Comparison results of emotions between 2 types of patients.

Group	Number of patients/case	SAS (points)		SDS (points)	
		Preoperative	Postoperative	Preoperative	Postoperative
Control group	41	56.21 ± 11.85	48.95 ± 10.26	58.34 ± 11.59	50.71 ± 9.24
Observers	41	55.93 ± 11.99	39.26 ± 9.11	58.19 ± 11.56	40.85 ± 8.11
*t* value	/	0.026	4.361	0.021	4.853
*P*-value	/	.871	.0156	.869	.0127

### 3.9. Satisfaction comparison results

**Figure 3.**
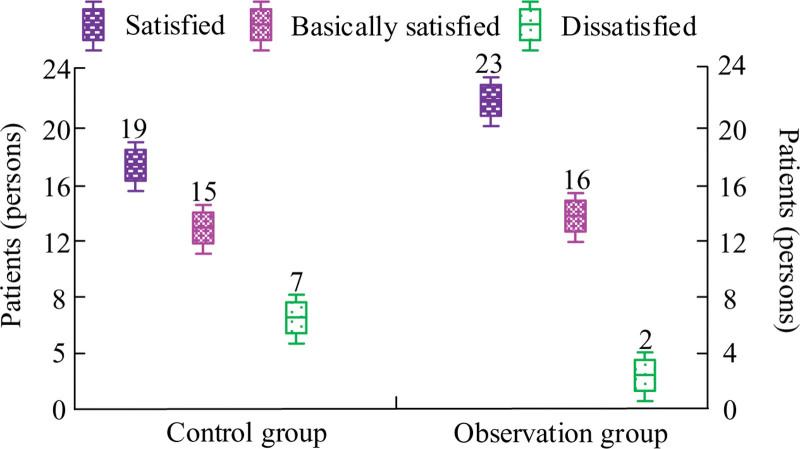
Comparison of patient satisfaction with postoperative care.

In terms of satisfaction, the overall satisfaction of OG patients was greatly superior than CG patients, with *P* < .05, as displayed in Figure 3.

## 4. Discussion and conclusion

Gastric cancer is a common malignant tumor that poses a huge threat to the health and safety of patients. Due to the lack of obvious early symptoms, many patients often enter the middle and late stages once discovered, which can bring huge pain to patients. What is even more concerning is that many patients may experience negative emotions such as F&A when facing such diseases. Long term physical exertion leads to impaired gastrointestinal function, resulting in malnutrition, weight loss, and decreased immune function. This situation seriously affects the quality of postoperative recovery, greatly reducing the patients’ living standard.^[16,17]^ At the same time, gastric cancer patients undergoing LRG-GC may experience incomplete gastrointestinal function due to the presence of drainage tubes and postoperative pain, although the surgical trauma is relatively small. This situation will undoubtedly affect the patient’s physical recovery and may even affect the effectiveness of the surgery. Therefore, postoperative care is particularly important for such patients. Traditional rehabilitation nursing strategies sometimes seem inadequate when faced with such situations. However, with the advancement of medicine and the increasing demand for care, the TPRN model has gradually received attention from people.^[18,19]^ This model combines 3 aspects of psychological, nutritional and sports nursing, aiming to improve patients’ gastrointestinal motility after surgery and promote the recovery of gastrointestinal function. It not only focuses on the physical health of patients, but also delves into their psychological level, providing comprehensive nursing services. Psychological care helps patients overcome F&A, and establish confidence in recovery. Nutritional care ensures that the nutritional needs of patients are met and enhances immunity. Sports care promotes the recovery of gastrointestinal function through appropriate exercise. Through extensive research and application, it has been found that the TPRN model is more conducive to improving the preoperative functional status of patients affected by laparoscopic surgery compared to conventional rehabilitation or a single rehabilitation strategy, promoting postoperative recovery of gastric cancer patients and reducing the incidence of postoperative complications.^[20]^ This indicates that the TPRN model has played a crucial role in caring the gastric cancer patients. It not only improves the quality of postoperative recovery for patients, but also reduces the incidence of complications. With the progress of medicine and the improvement of people’s demand for care, this model will be more widely used in clinical practice in the future. For medical staff, in-depth research and promotion of the TPRN model is of great significance. This can not only help more gastric cancer patients achieve better quality of life, but also a powerful practice of medical humanistic care.

In LRG-GC, the physical condition of patients during the perioperative period can timely reflect their postoperative recovery. By comparing perioperative indicators between 2 groups of patients, the FET, FGOBT, FBMT, and HT of CG patients were 78.26 ± 9.12, 29.83 ± 8.05, 3.98 ± 0.93, and 9.57 ± 1.49, respectively. The FET, FGOBT, FBMT, and HT of OG patients were 62.15 ± 5.93, 18.67 ± 7.55, 2.05 ± 0.58, and 7.21 ± 1.05, respectively. The data shows that in the TPRN mode, patients have a faster postoperative recovery progress, and HT is also significantly shortened, which can reduce the patient’s treatment expenses. The gastrointestinal symptoms of patients can be used to evaluate their postoperative recovery. This study will use the scores of gastrointestinal symptoms before and after nursing to evaluate the recovery of gastrointestinal function in patients. The GSS of CG and OG before nursing was 15.37 ± 2.75 and 15.13 ± 2.47, while after nursing it was 10.53 ± 2.66 and 8.01 ± 1.36. The results indicate that the TPRN mode can significantly reduce gastrointestinal discomfort and improve the recovery speed of patients. The immune indicators have important significance in the clinical efficacy study of TPRN mode on LRG-GC. By monitoring changes in immune indicators, we can better understand the patient’s immune status and surgical risks, develop more personalized nursing plans and rehabilitation plans, promote patient recovery, and improve surgical success rates. The results showed a significant decrease in IgG before and after CG surgery, while the postoperative IgG value of OG was higher than the preoperative value. In Patients who received preoperative exercise showed a significant decrease in preoperative and postoperative 6MWT in the CG, while the postoperative value of 6MWT in the OG was higher than the preoperative value. This indicates that triple pre rehabilitation nursing is more conducive to improving the patient’s preoperative and postoperative immune abilities, and promoting rapid recovery of postoperative immune function. By monitoring the levels of ALB and PAB, it is possible to better understand the nutritional status and protein metabolism of patients. This study tested the ALB and PAB of patients, and found that both the ALB and PAB of CG showed a certain decrease after surgery. The postoperative values of ALB for CG and OG were 31.59 ± 7.65 and 36.08 ± 8.27, respectively, while the postoperative values of PAB were 0.19 ± 0.05 and 0.27 ± 0.09, respectively. This indicates that the ALB and PAB values of OG in TPRN mode are significantly higher than those of CG, which can alleviate the nutritional problems of the body to a certain extent and effectively help patients recover their physical functions. In order to verify the effect of acupoint paste combined with ear acupoint bean pressing burial combined with triple pre-rehabilitation nursing in traditional Chinese medicine nursing in laparoscopic radical surgery for gastric cancer, the study took the recovery of gastrointestinal function as a comparative index, which was used to verify the clinical effect. The duration of abdominal pain, bloating, and bowel sound recovery in OG were 7.96 ± 1.58, 9.21 ± 2.68, and 18.26 ± 2.79, respectively, and the recovery time was significantly shorter than that in CG. This suggests that acupoint paste combined with acupoint buri bean therapy in TCM care can promote the recovery of bowel function after laparoscopy by regulating the release of gastrointestinal hormones,^[21]^ while the combination of triple prehabilitation care can enhance the physical recovery of postoperative patients. Due to factors such as pain, F&A, patients can affect their sleep quality, which not only affects their recovery progress but also leads to the occurrence of complications. Through effective statistics on the sleep quality of 82 patients, the sleep quality before CG nursing was 22.57 ± 3.66, and the sleep quality after nursing was 14.36 ± 3.72, which verified the value of TPRN after surgery. Through TPRN, medical staff have strengthened communication and interaction with patients. Effective psychological counseling can enhance patient confidence and alleviate their negative emotions. The experiment showed that the SAS and SDS scores after nursing were significantly lower than those of CG. This also confirms that TPRN can enhance the patient’s positive cooperation with treatment, laying a foundation for further promoting rapid recovery of postoperative physical function. The hospital conducts a patient satisfaction survey with the aim of understanding the patient’s evaluation and recognition of nursing services. Through investigation, hospitals can better identify shortcomings in their services, improve nursing services, increase patient satisfaction and rehabilitation outcomes. The survey on patient satisfaction showed that the satisfaction rate of OG was 56.10%, and the satisfaction rate of CG was 46.34%, indicating the importance of TPRN in LRG-GC. This study still has limitations. It is a retrospective study with selection bias and confounding bias. Secondly, the sample size of this study is relatively small, so it is necessary to increase the follow-up time for tumor patients to observe the long-term efficacy, but this does not affect the accuracy of this study. We plan to conduct a multi-center randomized controlled experiment in the future, and expand the time of recording and follow-up. Search for better TCM treatment.

In summary, implementing the TPRN model in the clinical treatment of LRG-GC can effectively improve the patient’s condition and nursing effectiveness, reduce the incidence of complications, and is worthy of further promotion and application in clinical practice.

## Author contributions

**Conceptualization:** Guangzhuo Ren, Liping Yang.

**Data curation:** Guangzhuo Ren, Liping Yang.

**Formal analysis:** Guangzhuo Ren, Liping Yang.

**Investigation:** Guangzhuo Ren, Liping Yang.

**Methodology:** Guangzhuo Ren, Liping Yang.

**Supervision:** Guangzhuo Ren, Liping Yang.

**Writing – original draft:** Guangzhuo Ren, Liping Yang.

**Writing – review & editing:** Guangzhuo Ren, Liping Yang.
